# *Vibrio phycocola* sp. nov. and *Vibrio phycohabitans* sp. nov., Isolated from the Phycosphere of Marine Algae

**DOI:** 10.4014/jmb.2604.04007

**Published:** 2026-05-04

**Authors:** Jeong Min Kim, Byeong Jun Choi, Hülya Bayburt, Jae Kyeong Lee, Ju Hye Baek, Baolei Jia, Che Ok Jeon

**Affiliations:** 1 Department of Life Science, Chung-Ang University, Seoul 06974, Republic of Korea; 2 Xianghu Laboratory, Hangzhou 311231, P. R. China

**Keywords:** *Vibrio phycocola*, *Vibrio phycohabitans*, New taxa, Marine algae, Phycosphere, Metabolic interaction, CAZy

## Abstract

Two Gram-stain-negative, facultatively aerobic, oxidase- and catalase-positive, motile (by means of a polar flagellum) rod-shaped bacterial strains, designated BS-M-Sm-2^T^ and MA40-2^T^, were isolated from marine algae. Growth was optimal at pH 7.0–8.0 and 2.0–3.0% (w/v) NaCl, with temperature optima of 25°C for BS-M-Sm-2^T^ and 25–30°C for MA40-2^T^. Ubiquinone-8 was the sole respiratory quinone. The major fatty acids common to both strains were C_16:0_, summed feature 3 (C_16:1_ *ω*7*c* and/or C_16:1_ *ω*6*c*), and summed feature 8 (C_18:1_ *ω*7*c* and/or C_18:1_ *ω*6*c*), while BS-M-Sm-2^T^ additionally contained C_12:0_ and C_14:0_. The predominant polar lipids were phosphatidylethanolamine and phosphatidylglycerol, with diphosphatidylglycerol also detected in strain MA40-2^T^. The DNA G+C contents of strains BS-M-Sm-2^T^ and MA40-2^T^ were 44.2 and 39.8 mol%, respectively. The 16S rRNA gene sequence similarity, average nucleotide identity (ANI), and digital DNA-DNA hybridization (dDDH) values between the two strains were 93.8%, 71.4%, and 23.2%, respectively. Phylogenetic and phylogenomic analyses placed both strains within the genus *Vibrio*, forming distinct lineages. Comparisons with closely related *Vibrio* type strains yielded ANI and dDDH values below 91.6% and 44.3%, respectively, further supporting their classification as novel species. Genome analyses revealed genes potentially involved in algal symbiosis, including those for polysaccharide degradation and vitamin biosynthesis. Based on comprehensive genomic, phylogenetic, phenotypic, and chemotaxonomic evidence, strains BS-M-Sm-2^T^ and MA40-2^T^ represent two novel species, for which the names *Vibrio phycocola* sp. nov. (BS-M-Sm-2^T^ =KACC 24066^T^ =DSM 119941^T^) and *Vibrio phycohabitans* sp. nov. (MA40-2^T^ =KACC 24064^T^ = DSM 119942^T^) are proposed.

## Introduction

The genus *Vibrio*, belonging to the family *Vibrionaceae*, comprises Gram-stain-negative, facultatively aerobic, motile, rod-shaped heterotrophic bacteria possessing a polar flagellum and typically exhibiting positive oxidase and catalase activities [1]. Historically, the genus has been closely associated with human pathogenicity due to the inclusion of the type species *Vibrio cholerae* and other well-known human pathogens, such as *Vibrio vulnificus*, *Vibrio parahaemolyticus*, and *Vibrio mimicus*. However, *Vibrio* encompasses a much broader ecological spectrum [2]. As of April 2026, more than 158 species have been validly published (LPSN), most of which have been isolated from diverse marine environments, including seawater, tidal sediments, marine organisms, and marine algae [3–10]. Although some species have been reported to be pathogenic to marine organisms, including marine algae [11, 12], the majority have not been associated with pathogenicity toward either humans or marine organisms.

*Vibrio* species are frequently associated with marine eukaryotes, including algae and corals, where they participate in complex symbiotic networks [2, 13]. In these environments, they contribute to key ecological processes, particularly nutrient cycling. For example, *Vibrio* species are often involved in the degradation of algal polysaccharides, such as alginate, laminarin, and carrageenan, thereby facilitating carbon turnover and nutrient recycling [14]. In addition, certain *Vibrio* strains produce essential growth factors, including B vitamins and phytohormone-like compounds, which can promote algal growth and development [15]. They may also enhance host fitness by producing antimicrobial compounds or by competitively excluding opportunistic pathogens [16], thereby contributing to the stability and resilience of algal-associated microbial communities [17, 18]. Collectively, these features highlight the ecological and functional importance of *Vibrio* species in marine ecosystems.

The surface of algae, known as the phycosphere, represents a dynamic microenvironment inhabited by complex bacterial assemblages that have co-evolved with their hosts [19, 20]. These associated microbes play critical roles in algal physiology by supplying essential nutrients and growth factors, such as vitamins and phenylacetate, fixing nitrogen, producing iron-storage proteins (*e.g.*, bacterioferritin), and protecting the host against pathogenic invasion [21-23]. In the course of investigating metabolic and symbiotic interactions between marine algae and their associated microbiota, we isolated several bacterial strains from the marine phycosphere [24-28]. In the present study, two strains presumed to belong to the genus *Vibrio* were isolated from the phycosphere of marine algae and were characterized using a polyphasic taxonomic approach, together with bioinformatic analyses to assess their potential metabolic interactions with marine algal hosts.

## Materials and Methods

### Isolation of Bacterial Strains

Strains BS-M-Sm-2^T^ and MA40-2^T^ were isolated from the phycosphere of two marine algae, the brown alga *Sargassum miyabei* collected from Mongdol Beach (34°44′28.9″N, 128°02′53.7″E) in April 2023 and the red alga *Grateloupia divaricata* collected from Moonam Beach (38°17′48.8″N, 128°32′59.1″E) in July 2021, respectively, in the Republic of Korea, as previously described [29]. Briefly, algal tissues were thoroughly washed with artificial seawater (ASW; 20.0 g NaCl, 2.9 g MgSO_4_, 4.5 g MgCl_2_·6H_2_O, 0.6 g KCl, and 1.8 g CaCl_2_·2H_2_O per liter) using a vortex mixer to remove loosely attached surface microorganisms. The washed tissues were then mechanically homogenized, serially diluted in ASW, and spread onto marine agar (MA; MBcell, Republic of Korea). Plates were incubated aerobically at 25°C for 5 days, after which emerging colonies were screened by PCR amplification of the 16S rRNA gene using the universal primers 27F (5′-AGA GTT TGA TCM TGG CTC AG-3′) and 1492R (5′-TAC GGY TAC CTT GTT ACG ACT T-3′), followed by restriction fragment analysis with HaeIII and HhaI and electrophoresis on 2% (w/v) agarose gels [29]. Representative amplicons exhibiting distinct fragment patterns were partially sequenced using primer 340F (5′-CCT ACG GGA GGC AGC AG-3′).

The obtained sequences were compared with those of validly published type strains using the EzBioCloud database [30], leading to the selection of two putative novel strains, BS-M-Sm-2^T^ and MA40-2^T^, for further taxonomic characterization. Both strains were routinely cultivated on MA at 25°C for 2 days and preserved at -80°C in marine broth (MB; MBcell) supplemented with 15% (v/v) glycerol.

### Phylogenetic Analysis Based on 16S rRNA Gene Sequences and Assessment of Ecological Distribution

The nearly full-length 16S rRNA gene sequences of strains BS-M-Sm-2^T^ and MA40-2^T^, initially amplified using primers 27F and 1492R, were further sequenced with the universal primers 518R (5′-ATT ACC GCG GCT GCT GG-3′) and 805F (5′-GAT TAG ATA CCC TGG TAG TC-3′) [29]. Sequences obtained using primers 340F, 518R, and 805F were assembled, and their similarities to those of closely related type strains were evaluated using the EzBioCloud platform. Multiple sequence alignments were performed using Infernal software (v1.1.4) [31]. Phylogenetic trees were reconstructed using the maximum-likelihood (ML), neighbor-joining (NJ), and maximum-parsimony (MP) methods implemented in MEGA11 [32], which were performed based on the Kimura two-parameter model, the nearest-neighbor interchange heuristic search, and the complete deletion option, respectively, with bootstrap support assessed from 1,000 replicates.

The potential ecological distributions of strains BS-M-Sm-2^T^ and MA40-2^T^ were assessed by comparing their 16S rRNA gene sequences against 500,048 publicly available metagenomic 16S rRNA amplicon datasets from diverse global environments, including marine and freshwater habitats, terrestrial soils, sediments, algae, invertebrates, and gut microbiomes, using the Integrated Microbial Next-Generation Sequencing (IMNGS) platform [33] with a sequence similarity threshold of 99.0%.

### Whole-Genome Sequencing, Genome-Based Phylogenetic and Relatedness Analyses, and Functional Genomic Analyses

Genomic DNA of strains BS-M-Sm-2^T^ and MA40-2^T^ was extracted from cells cultured in MB using the Wizard Genomic DNA Purification Kit (Promega, USA) according to the manufacturer’s instructions. The genome of strain BS-M-Sm-2^T^ was sequenced using a hybrid approach that combined the Illumina HiSeq X platform (151 bp paired-end reads; Illumina, USA) and the Oxford Nanopore MinION platform (Oxford Nanopore Technologies, UK). Nanopore long reads were assembled *de novo* using Flye (v2.9.3) [34], and the resulting contigs were iteratively polished with Illumina reads using Pilon (v1.24) [35] until no further corrections were required. In contrast, the genome of strain MA40-2^T^ was sequenced solely using the MinION platform, and the long reads were assembled *de novo* with Flye. Genome quality, including completeness and contamination, was assessed using CheckM2 (v1.1.0) [36].

Phylogenomic relationships were inferred based on the concatenated amino acid sequences of 120 universal single-copy marker genes (bac120 set) using the Genome Taxonomy Database Toolkit (GTDB-Tk; v2.4.1) [37]. An ML phylogenomic tree was reconstructed, and its robustness was evaluated using 1,000 bootstrap replicates. The resulting tree was visualized and refined using MEGA11. Genome relatedness between strains BS-M-Sm-2^T^ and MA40-2^T^ and their closest type strains was assessed by calculating average nucleotide identity (ANI) using the Orthologous Average Nucleotide Identity Tool (OAT, v0.93.1) [38] and digital DNA-DNA hybridization (dDDH) values using the Genome-to-Genome Distance Calculator (GGDC, v3.0; formula 2) [39].

The whole-genome sequences of strains BS-M-Sm-2^T^ and MA40-2^T^ were deposited in GenBank, and gene annotation was performed using the NCBI Prokaryotic Genome Annotation Pipeline (PGAP). Functional annotation of genes potentially involved in algal-bacterial interactions was conducted against the KEGG Orthology database using the BlastKOALA server [40]. Carbohydrate-active enzymes (CAZys) were identified and annotated using the dbCAN3 server integrated with the CAZy database [41]. In addition, secondary metabolite biosynthetic gene clusters (BGCs) were predicted to use antiSMASH bacterial version 8.0 with default parameters [42]. Genes associated with virulence factors (VFs) were identified by BLASTX searches against the Virulence Factor Database (VFDB) [43] accessible via ABRicate (v1.0.1, https://github.com/tseemann/abricate), with thresholds of >50% sequence identity and >80% coverage.

### Analysis of Phenotypic and Biochemical Characteristics

Growth characteristics of strains BS-M-Sm-2^T^ and MA40-2^T^ were examined on various agar media, including MA, Reasoner’s 2A (R2A) agar, Luria-Bertani (LB) agar, tryptic soy agar (TSA), and nutrient agar (NA) (all from MBcell). Growth ranges were determined on MA at temperatures from 5 to 45°C (in 5°C intervals) and in MB at pH 4.0–11.0 (in 1.0-unit intervals) after 2 days of incubation. For pH experiments, MB was buffered with 0.05 M sodium citrate (pH 4.0–5.0), sodium phosphate (pH 6.0–8.0), or sodium carbonate–bicarbonate (pH 9.0–11.0), and the final pH was adjusted after autoclaving when necessary. Salt tolerance was assessed in MB containing 0–15% (w/v) NaCl (in 1.0% increments), prepared by modifying the NaCl concentration of the MB formulation.

Anaerobic growth was evaluated by incubating cultures on MA at 25°C for 21 days in a GasPak Plus system (BBL, USA). Cell morphology and motility were observed using a phase-contrast microscope (Axio Scope.A1; Carl Zeiss, Germany) with cells grown on MA at 25°C for 2 days. For detailed examination of cell structure and flagellation, cells were negatively stained with 2% (w/v) uranyl acetate on Formvar-coated copper grids and observed using a transmission electron microscope (JEM-1010; JEOL, Japan). Gliding motility was assessed on MA supplemented with 0.3% (w/v) agar [44]. Gram staining was performed using a commercial kit (bioMérieux, France) according to the manufacturer’s instructions.

Oxidase activity was determined using 1% (w/v) tetramethyl-*p*-phenylenediamine (Merck, USA), and catalase activity was assessed by bubble formation in 3% (v/v) hydrogen peroxide. Hydrolytic activities toward casein (1% skim milk), esculin (0.1%), starch (1%), L-tyrosine (0.5%), Tween 20 (1%), and Tween 80 (1%) were tested on MA following standard procedures [45]. Additional biochemical characteristics were determined using the API 20NE system (bioMérieux) with inocula prepared in ASW according to the manufacturer’s instructions.

### Analysis of Chemotaxonomic Characteristics

Respiratory isoprenoid quinones of strains BS-M-Sm-2^T^ and MA40-2^T^ were extracted from cells grown in MB according to Minnikin *et al*. [46] and analyzed by high-performance liquid chromatography (HPLC; LC-20A, Shimadzu, Japan) using a reversed-phase Kromasil column (250 × 4.6 mm; Akzo Nobel Center, Netherlands) equipped with a diode-array detector (SPD-M20A, Shimadzu). Methanol-isopropanol (2:1, v/v) was used as the mobile phase under isocratic conditions at a flow rate of 1 mL/min.

For cellular fatty acid analysis, strains BS-M-Sm-2^T^ and MA40-2^T^, along with four reference strains, were cultivated aerobically in MB at their respective optimal temperatures. Cells were harvested during the exponential growth phase (OD_600_ = 0.7–0.8). Fatty acids were saponified, methylated, and extracted following the standard MIDI protocol. The resulting fatty acid methyl esters were analyzed using a Hewlett Packard 6890 gas chromatograph and identified with the RTSBA6 database in the Sherlock Microbial Identification System (v6.0B) [47].

Polar lipids were extracted and separated by two-dimensional thin-layer chromatography (TLC) as described by Minnikin *et al*. [46]. Lipid classes were visualized using specific detection reagents: 10% ethanolic molybdophosphoric acid for total polar lipids, ninhydrin for aminolipids, Dittmer-Lester reagent for phospholipids, and α-naphthol/sulfuric acid for glycolipids. The presence of phosphatidylglycerol (PG), phosphatidylethanolamine (PE), and diphosphatidylglycerol (DPG) was confirmed using standard compounds (Sigma-Aldrich, USA).

## Results and Discussion

### Isolation and 16S rRNA Gene Sequence-Based Phylogenetic Characteristics of Strains BS-M-Sm-2^T^ and MA40-2^T^

To avoid the repeated isolation of identical bacterial strains from marine algae, colonies grown on MA were subjected to PCR amplification of the 16S rRNA gene, followed by restriction fragment analysis, and only colonies exhibiting distinct fragment patterns were selected for sequencing, resulting in the isolation of two putative novel strains, BS-M-Sm-2^T^ and MA40-2^T^, affiliated with the genus *Vibrio*. In addition, nearly complete 16S rRNA gene sequences were obtained for strain BS-M-Sm-2^T^ (1,555 bp) and strain MA40-2^T^ (1,476 bp) by assembling reads generated using the universal primers 340F, 518R, and 805F.

The sequence similarity between the two strains was 93.8%, which is well below the accepted species delineation threshold of 98.5–98.7% [48], indicating that they represent distinct species. Strain MA40-2^T^ showed the highest sequence similarity to *Vibrio algarum* KJ40-1^T^ (96.7%), followed by *Vibrio variabilis* R-40492^T^ (95.3%) and *Vibrio ulleungensis* 188UL20-2^T^ (95.2%), supporting its status as a novel species. In contrast, strain BS-M-Sm-2^T^ exhibited high sequence similarity to *Vibrio crassostreae* LGP7^T^ (99.8%), *Vibrio pomeroyi* LMG 20537^T^ (99.7%), and *Vibrio gigantis* CAIM 25^T^ (99.6%), indicating that 16S rRNA gene sequence similarity alone is insufficient to determine its taxonomic status at the species level.

**Fig. 1:**
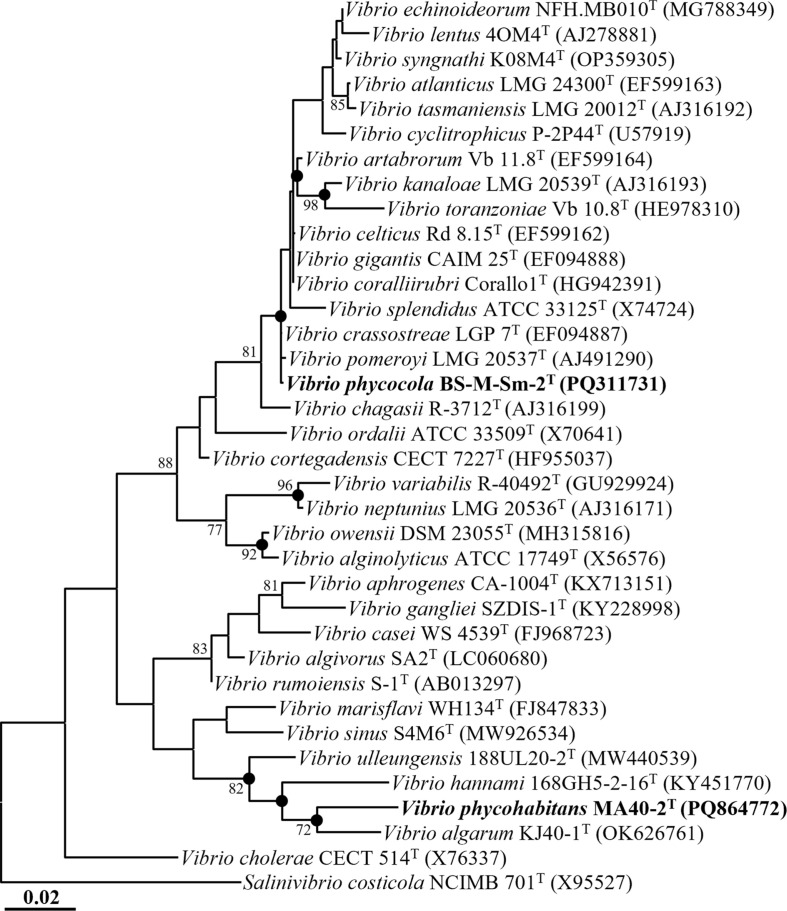
Maximum-likelihood tree showing the phylogenetic relationships of strains BS-M-Sm-2^T^ and MA40-2^T^ and their closely related type strains, based on 16S rRNA gene sequences. Bootstrap support values (>70%) are indicated at the nodes as percentages derived from 1,000 replicates. Filled circles ( ● ) denote nodes that were also retrieved in the trees constructed using the neighbor-joining and maximum-parsimony algorithms. *Salinivibrio costicola* NCIMB 701^T^ (X95527) was used as the outgroup. Scale bar represents 0.02 nucleotide substitutions per site.

Phylogenetic analysis based on 16S rRNA gene sequences using the ML method showed that strain BS-M-Sm-2^T^ formed a clade with *V. pomeroyi* LMG 20537?, *V. chagasii* R-3712^T^, and *V. crassostreae* LGP7^T^, whereas strain MA40-2^T^ clustered with *V. algarum* KJ40-1^T^, and both strains were clearly positioned within the genus *Vibrio* (Fig. 1). Nearly identical topologies were observed in the NJ and MP trees (Fig. S1). Collectively, these phylogenetic and sequence similarity analyses based on 16S rRNA gene sequences support the assignment of strains BS-M-Sm-2^T^ and MA40-2^T^ to the genus *Vibrio* and indicate that they represent two distinct species.

### Ecological Insights into Strains BS-M-Sm-2^T^ and MA40-2^T^ Based on their Distribution

Ecological habitat distribution analysis, based on comparisons of 16S rRNA gene sequences against diverse environmental datasets, revealed that strains BS-M-Sm-2^T^ and MA40-2^T^ exhibit markedly distinct distribution patterns in natural environments, despite both being isolated from marine algae (Table S1). Strain BS-M-Sm-2^T^ showed a broad and relatively high prevalence across diverse marine habitats, including marine animals (*e.g.*, *Crassostrea gigas*, *Eunicella cavolini*, *Litoditis marina*, *Tripneustes gratilla*, *Eunicella singularis*, and oysters), as well as corals, algae, fish, and seawater. In contrast, strain MA40-2^T^ exhibited a much more restricted distribution, being primarily detected in a limited number of hosts and environments, such as *Tripneustes gratilla*, *Centricnemus leucogrammus*, and other invertebrates. Notably, strain MA40-2^T^ was absent from most habitats in which strain BS-M-Sm-2^T^ was identified, indicating that the two strains occupy substantially different ecological niches.

The distribution patterns further suggest that, although both strains were isolated from algal phycospheres, marine animals may represent more important environmental reservoirs. Interestingly, strain MA40-2^T^, but not BS-M-Sm-2^T^, was also detected in non-marine environments, including terrestrial insects (*e.g.*, *Centricnemus leucogrammus* and *Crioceris duodecimpunctata*) and plants such as tomato (*Solanum lycopersicum*). Overall, these results indicate that strain BS-M-Sm-2^T^ is broadly distributed as a generalist across marine environments, particularly in association with marine animals, whereas strain MA40-2^T^ appears to occupy a more specialized ecological niche spanning limited marine and certain terrestrial environments.

### Genome-Based Phylogeny, Relatedness, and Genomic Features of Strains BS-M-Sm-2^T^ and MA40-2^T^

*De novo* assembly of sequencing reads generated complete genomes for strains BS-M-Sm-2^T^ and MA40-2^T^, with genome sizes of approximately 5,430 kb and 4,593 kb and average coverages of 707.0× and 72.0×, respectively. The 16S rRNA gene sequences retrieved from the assembled genomes were identical to those obtained by PCR-based sequencing. Genome quality assessment using CheckM2 indicated high assembly reliability, with 100% completeness for both genomes and minimal contamination levels (0.00% for strain BS-M-Sm-2^T^ and 0.07% for strain MA40-2^T^), meeting the criteria for ‘trusted’ genomes (≥90% completeness and ≤10% contamination) [36].

**Fig. 2:**
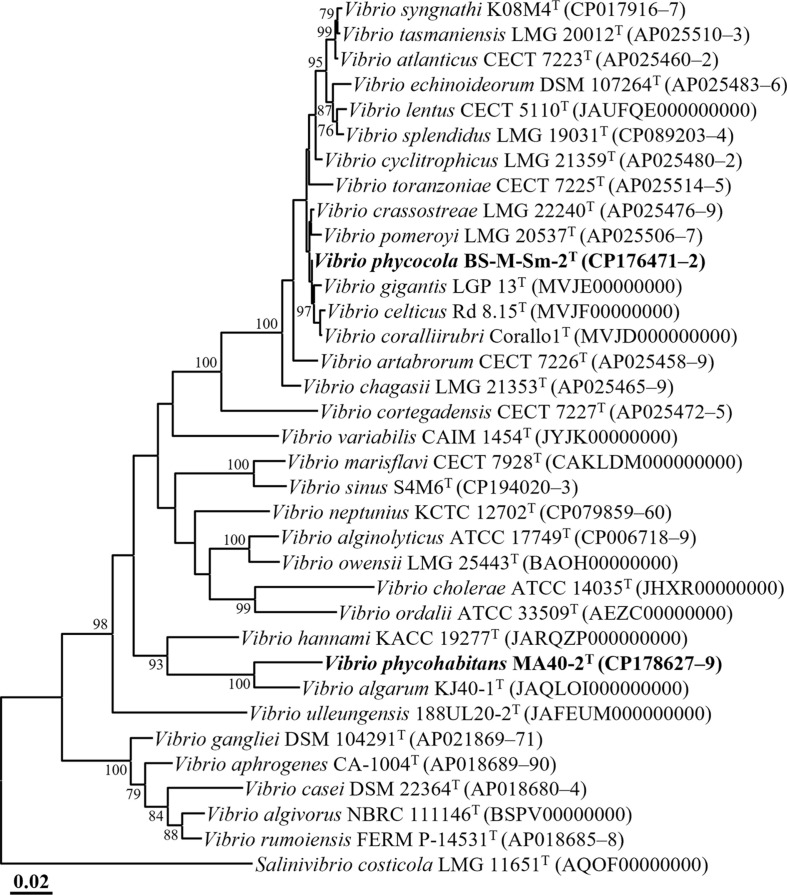
Phylogenomic tree showing the phylogenetic relationships of strains BS-M-Sm-2^T^ and MA40-2^T^ and their closely related type strains, based on the concatenated protein sequences of 120 single-copy marker genes (bac120 marker set) of GTDB-Tk. Bootstrap support values (>70%) are indicated at the nodes as percentages derived from 1,000 replicates. *Salinivibrio costicola* LMG 11651^T^ (AQOF00000000) was used as the outgroup. Scale bar represents 0.02 substitutions per amino acid.

An ML phylogenomic tree based on the concatenated amino acid sequences of 120 conserved marker genes showed that strains BS-M-Sm-2^T^ and MA40-2^T^ formed distinct and well-supported lineages within the genus *Vibrio* (Fig. 2), consistent with the 16S rRNA gene-based phylogenies (Figs. 1 and S1). Strain BS-M-Sm-2^T^ formed a separate lineage from *V. gigantis* LGP 13^T^, *V. celticus* Rd 8.15^T^, and *V. coralliirubri* Corallo1^T^, whereas strain MA40-2^T^ clustered with *V. algarum* KJ40-1^T^ with strong bootstrap support (100%). Based on both 16S rRNA gene sequence and genome-based phylogenetic analyses, *V. algarum* KACC 22588^T^, *V. hannami* KACC 19277^T^, *V. crassostreae* LMG 22240^T^, and *V. gigantis* LMG 22741^T^ were selected as reference strains for subsequent comparisons of phenotypic characteristics and fatty acid compositions.

Genome relatedness analyses further supported the distinct taxonomic status of the two strains. The ANI and dDDH values between strains BS-M-Sm-2^T^ and MA40-2^T^ were 71.4% and 23.2%, respectively, which are well below the accepted thresholds for species delineation (~95% ANI and 70% dDDH) [48]. Similarly, ANI and dDDH values between each strain and their closest type strains were also below these thresholds (≤91.6% ANI and ≤44.3% dDDH for BS-M-Sm-2^T^; ≤75.6% ANI and ≤22.9% dDDH for MA40-2^T^; Table S2). Collectively, the phylogenomic and genome relatedness analyses suggest that both strains represent novel species within the genus *Vibrio*.

The genome of strain BS-M-Sm-2^T^ consists of two circular chromosomes of 3,565 kb (44.5 mol% G+C content) and 1,865 kb (44.0 mol% G+C content), encoding a total of 4,819 genes, including 4,576 protein-coding sequences (CDSs), 43 rRNA genes, 138 tRNA genes, and 4 non-coding RNA genes. Similarly, the genome of strain MA40-2^T^ comprises two circular chromosomes of 2,869 kb and 1,678 kb (both with 39.5 mol% G+C content), as well as a 45.0 kb plasmid (45.0 mol% G+C content), collectively encoding 4,136 genes, including 3,941 CDSs, 25 rRNA genes, 92 tRNA genes, and 4 non-coding RNA genes. The average genomic DNA G+C contents were 44.2 mol% for BS-M-Sm-2^T^ and 39.8 mol% for MA40-2^T^, values that are consistent with those reported for members of the genus *Vibrio*. A comprehensive summary and comparison of genomic features with those of closely related type strains are provided in Table 1.

### Polysaccharide Degradation Potentials of Strains BS-M-Sm-2^T^ and MA40-2^T^

Given that phycosphere-associated bacteria are characterized by their ability to utilize algal cell wall-derived polysaccharides as primary carbon sources [49], we examined the CAZy repertoires of strains BS-M-Sm-2^T^ and MA40-2^T^, along with those of their closely related type strains (Table 1). The genomes of strains BS-M-Sm-2^T^ and MA40-2^T^ were predicted to encode 84 and 67 CAZy genes, respectively. These genes were classified into six functional categories: glycoside hydrolases (GH; 45 and 33 genes), glycosyltransferases (GT; 23 and 20 genes), polysaccharide lyases (PL; 3 and 8 genes), carbohydrate esterases (CE; 5 and 2 genes), carbohydrate-binding modules (CBM; 5 and 4 genes), and auxiliary activities (AA; 3 and 0 genes).

**Fig. 3:**
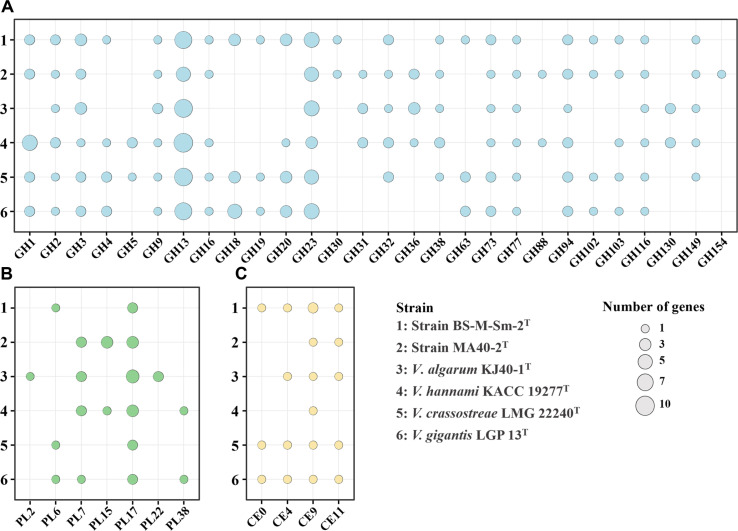
Bubble plots showing the distribution of carbohydrate-active enzyme (CAZyme) genes in strains BS-M-Sm-2^T^ and MA40-2^T^, together with their closely related type strains. CAZyme genes are classified into glycoside hydrolase (GH; A), polysaccharide lyase (PL; B), and carbohydrate esterase (CE; C) families. Bubble size represents the number of genes assigned to each CAZy family.

Although the total numbers of CAZy genes, particularly those directly involved in polysaccharide depolymerization (GH, PL, and CE), were generally comparable to those of closely related type strains, clear differences were observed at the level of specific enzyme families (Fig. 3), indicating variation in carbohydrate-degrading capacities. All strains commonly possessed GH2, GH3, GH9, GH13, GH23, GH73, GH77, GH94, GH116, PL17, and CE9, suggesting a shared ability to degrade major polysaccharides such as starch, laminarin, alginate, and peptidoglycan. In contrast, variation was observed in several CAZyme families, including GH4, GH18, GH19, GH20, GH63, PL2, PL6, CE0, and CE4. These differences indicate that the strains differ in their capacity to degrade structurally diverse polysaccharides. In particular, the presence or absence of chitinolytic enzymes (GH18, GH19, GH20, and CE4) suggests differential abilities to utilize chitin and related amino sugar polymers, whereas variation in PL families (PL2, PL6, and PL15) implies differences in substrate specificity toward uronic acid-rich polysaccharides such as alginate and pectin-like compounds. Additionally, the occurrence of GH63 and GH4 suggests variability in the utilization of glycoproteins and modified carbohydrates. Collectively, these results indicate that while strains BS-M-Sm-2^T^ and MA40-2^T^ share a common capacity to degrade key algal polysaccharides, they exhibit functional differentiation in the utilization of more structurally complex or specialized substrates, which likely contributes to ecological niche partitioning within the phycosphere.

**Fig. 4:**
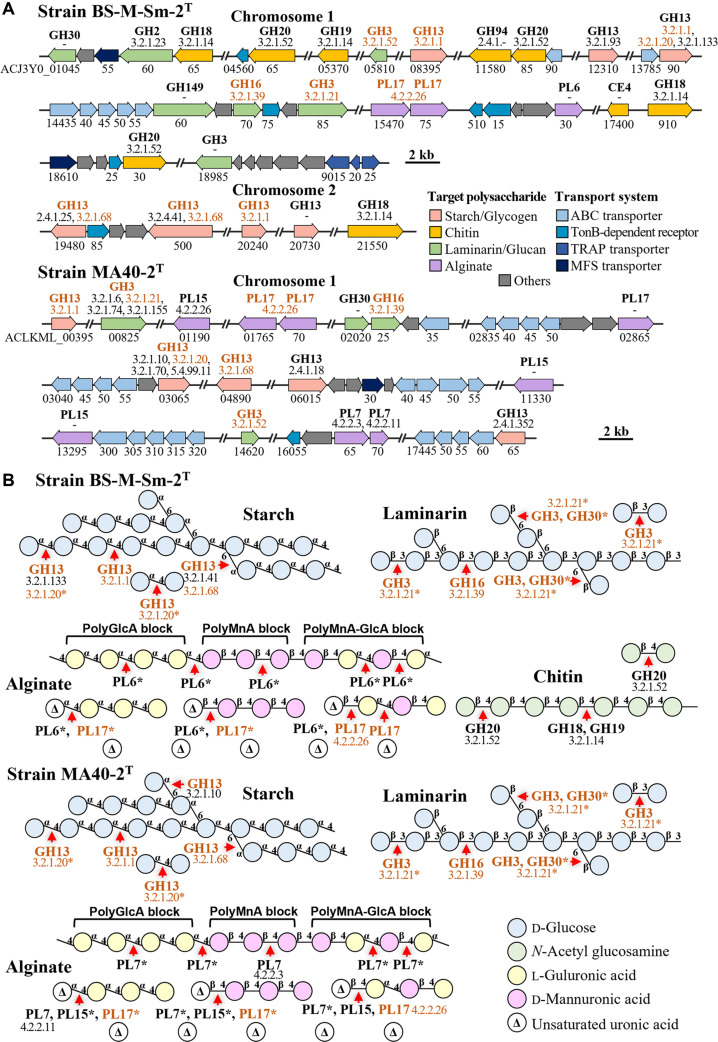
Genomic organization of carbohydrate-active enzyme (CAZyme) genes involved in the degradation of starch, laminarin, alginate, and chitin, along with their neighboring genes, in the genomes of strains BS-M-Sm-2^T^ and MA40-2^T^ (A), and the predicted cleavage sites of these CAZymes on the respective polysaccharides (B). Genes shared by both strains are highlighted in brown. Genes with ambiguous target sites are marked with an asterisk (*).

To further investigate their polysaccharide metabolic potential, we examined the genomic organization of CAZyme genes associated with the degradation of starch, laminarin, alginate, and chitin (Fig. 4A). Notably, genes involved in the degradation of individual polysaccharides were generally dispersed throughout the genome rather than organized into distinct operons or tightly clustered regions. This scattered distribution suggests that strains BS-M-Sm-2^T^ and MA40-2^T^ are not specialized for the utilization of a single polysaccharide but are instead adapted to dynamic environments containing diverse and fluctuating polysaccharide resources, potentially through gene gain and loss events. Furthermore, the predicted cleavage sites of CAZymes acting on these polysaccharides were mapped to illustrate and compare their potential degradation pathways in both strains (Fig. 4B).

Both strains possessed a conserved set of CAZyme genes required for the degradation of starch and laminarin, indicating their ability to hydrolyze these polysaccharides into mono- or oligosaccharides. Although the two strains harbored different CAZyme complements associated with alginate degradation, their predicted cleavage sites were similar, suggesting comparable initial depolymerization processes. However, while both strains appear capable of degrading alginate into unsaturated uronic acid intermediates, they may lack the complete enzymatic pathways required for further conversion into monomeric compounds. In contrast, the two strains differed markedly in their potential for chitin degradation. Strain BS-M-Sm-2^T^ possessed a complete set of CAZyme genes involved in chitin depolymerization, including GH18 (chitinases), GH19 (chitinases), GH20 (*β*-*N*-acetylhexosaminidase), and CE4 (chitin deacetylase), whereas these genes were absent in strain MA40-2^T^. These results indicate that strain BS-M-Sm-2^T^ has a substantially greater capacity to utilize chitin-derived substrates than strain MA40-2^T^. This enhanced metabolic versatility may be associated with the broader environmental distribution of strain BS-M-Sm-2^T^ compared with MA40-2^T^ (Table S1).

### Genomic Analysis of Potential Genes associated with Interactions with Algal Hosts

**Fig. 5:**
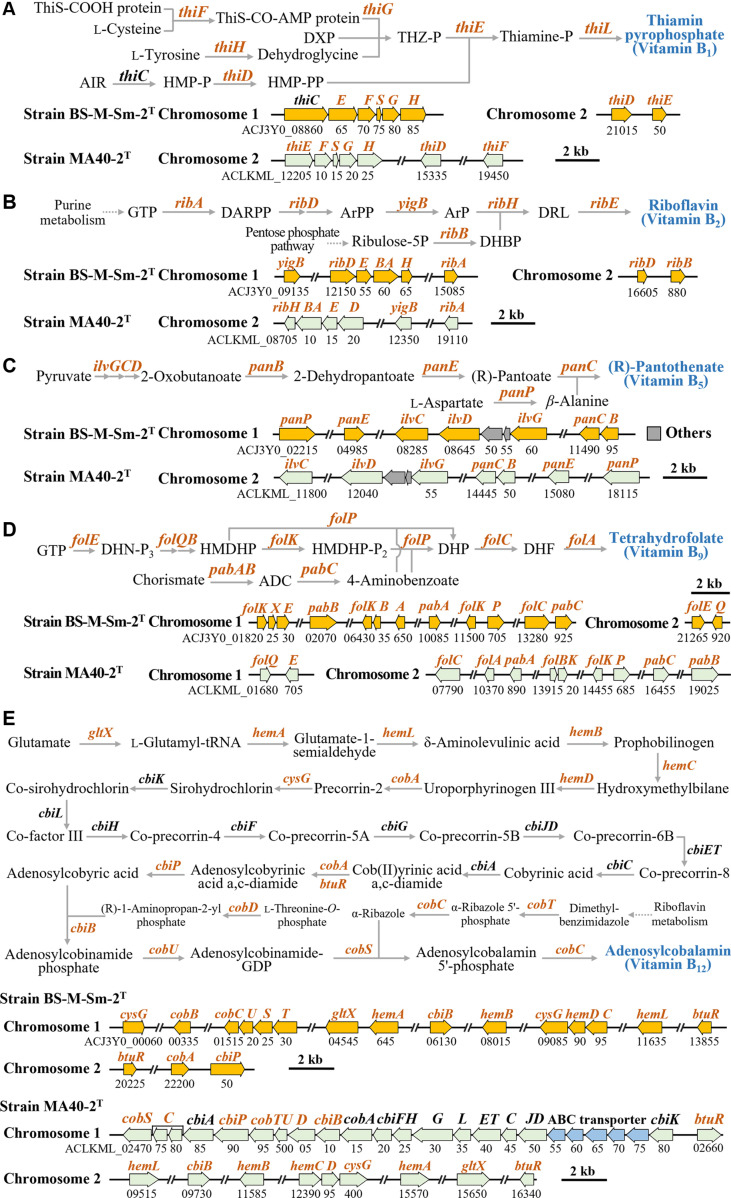
Biosynthetic pathways and genomic organization of thiamin pyrophosphate (vitamin B_1_; A), riboflavin (vitamin B_2_; B), (R)-pantothenate (vitamin B_5_; C), tetrahydrofolate (vitamin B_9_; D), and adenosylcobalamin (vitamin B_12_; E) identified in strains BS-M-Sm-2^T^ and MA40-2^T^. Genes shared by both strains are highlighted in brown.

Bacteria inhabiting the algal phycosphere can support the growth of algal hosts through various metabolic interactions, including the production and provision of essential compounds such as vitamins and nitrogen compounds [22, 23]. Genomic analyses showed that strains BS-M-Sm-2^T^ and MA40-2^T^ possess genes associated with the biosynthesis of several B vitamins, including thiamin (B_1_), riboflavin (B_2_), pantothenate (B_5_), folate (B_9_), and cobalamin (B_12_) (Fig. 5), suggesting their potential to supplement these nutrients to marine algae that lack complete biosynthetic pathways. Both strains possess complete gene sets for the biosynthesis of riboflavin, pantothenate, and folate. However, strain MA40-2^T^ lacks *thiC* (encoding 4-amino-5-hydroxymethyl-2-methylpyrimidine phosphate synthase), a key enzyme in thiamin biosynthesis, whereas strain BS-M-Sm-2^T^ lacks several essential genes required for cobalamin biosynthesis, indicating that these strains are likely unable to independently synthesize thiamin and cobalamin, respectively. Nevertheless, such partial deficiencies do not preclude ecological functionality. In natural phycosphere communities, metabolic complementarity among coexisting microorganisms may enable cooperative vitamin production [50]. Thus, strains BS-M-Sm-2^T^ and MA40-2^T^ may still contribute to vitamin availability through shared or cross-feeding metabolic networks within the algal-associated microbiome.

**Fig. 6:**
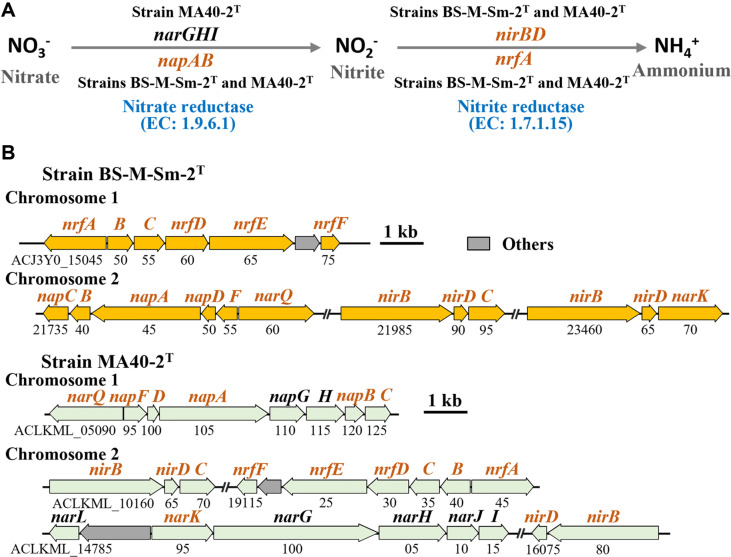
Metabolic pathway of dissimilatory nitrate reduction to ammonium (DNRA) (A) and the corresponding DNRA-associated genes (B) identified in strains BS-M-Sm-2^T^ and MA40-2^T^. Genes shared by both strains are highlighted in brown. *nrfA*, ammonia-forming nitrite reductase cytochrome c552 subunit; *nrfB*, cytochrome c nitrite reductase pentaheme subunit; *nrfC*, cytochrome c nitrite reductase Fe-S protein; *nrfD*, cytochrome c nitrite reductase subunit; *nrfE* and *nrfF*, heme lyase family subunit; *nirB*, nitrite reductase large subunit; *nirC*, formate/nitrite transporter family protein; *nirD*, nitrite reductase small subunit; *napA*, periplasmic nitrate reductase subunit alpha; *napB*, nitrate reductase cytochrome c-type subunit; *napC*, NapC/NirT family cytochrome c; *napD*, chaperone NapD; *napF*, ferredoxin-type protein NapF; *narK*, NarK family nitrate/nitrite MFS transporter; *narQ*, nitrate/nitrite twocomponent system sensor; *narL*, two-component system response regulator; *narG*, nitrate reductase subunit alpha; *narH*, nitrate reductase subunit beta; *narJ*, nitrate reductase molybdenum cofactor assembly chaperone; *narI*, respiratory nitrate reductase subunit gamma.

Notably, both strains encode complete gene sets for dissimilatory nitrate reduction to ammonium (DNRA) (Fig. 6), indicating their potential to use nitrate as a terminal electron acceptor under microaerobic or anaerobic conditions and to produce ammonium as the end product. In contrast to denitrification, which converts nitrate into nitrogen gas and results in the loss of bioavailable nitrogen, DNRA retains nitrogen within the ecosystem by reducing nitrate to ammonium. This process not only prevents nitrogen loss but may also limit competing nitrate reduction pathways by other microorganisms. Consequently, the production of ammonium via DNRA represents an efficient nitrogen-retention and sharing mechanism that can enhance nitrogen availability for algal hosts, thereby supporting primary production in nitrogen-limited marine environments [51]. Although both strains possess the core genetic components required for DNRA, strain MA40-2^T^ additionally harbors genes such as *narGHI* and *napGH*, which are associated with nitrate reduction processes. This expanded gene repertoire suggests that strain MA40-2^T^ may have a greater capacity or flexibility for nitrate reduction and, potentially, a more efficient DNRA process compared with strain BS-M-Sm-2^T^.

Furthermore, strain BS-M-Sm-2^T^ harbors genes encoding arginine decarboxylase (*speA*) and agmatinase (*speB*), which catalyze the conversion of arginine to putrescine, as well as aromatic amino acid aminotransferase (*hisC*) and 4-hydroxyphenylpyruvate dioxygenase (*hppD*), which are involved in the production of the auxin-like compound 2-hydroxyphenylacetic acid. These metabolites are known to mitigate abiotic stress and promote algal growth [22]. Both strains also possess BGCs for arylpolyenes and terpenes, which are associated with cellular protection. However, their adaptive strategies differ: strain BS-M-Sm-2^T^ encodes a BGC for a Ni-type siderophore, suggesting specialized iron acquisition, as well as a RiPP-like cluster with potential antimicrobial activity, whereas strain MA40-2^T^ harbors a complete ectoine biosynthetic gene cluster, indicative of enhanced osmoprotection.

Because the genus *Vibrio* includes several well-known pathogens, we further examined the genomes of strains BS-M-Sm-2^T^ and MA40-2^T^ for virulence-associated genes based on VFDB. No genes encoding major exotoxins reported in pathogenic *Vibrio* species, including *ctxAB*, *tdh*, *vvhA*, *zot*, and *tlh* [52], were detected (Table S3). In addition, no genes associated with toxin secretion or delivery systems were identified. These exotoxin-related genes were likewise absent from all reference strains used for comparison in this study, including strains isolated from oysters. However, genes involved in general protein secretion (*eps* operon; type II secretion system), surface adherence (*ompU/mam7*), and motility (*flg*, *fli*, and *che*), which are broadly distributed among environmental vibrios and are not directly indicative of pathogenicity, were detected. Collectively, these findings suggest that strains BS-M-Sm-2^T^ and MA40-2^T^ lack known major virulence determinants and are more likely to function as beneficial algal symbionts than as pathogens.

### Phenotypic and Biochemical Characteristics

Both strains BS-M-Sm-2^T^ and MA40-2^T^ exhibited robust growth on MA and showed good growth on R2A agar, TSA, and NA supplemented with 2% (w/v) NaCl, whereas only weak growth was observed on LB agar containing 2% NaCl. Cells of both strains were Gram-stain-negative, motile rods. Strain BS-M-Sm-2^T^ measured approximately 0.5–0.7 μm in width and 1.8–2.2 μm in length, whereas strain MA40-2^T^ measured 0.4–0.6 μm in width and 1.5–1.8 μm in length (Fig. S2). Both strains exhibited slight growth on MA under anaerobic conditions after 21 days of incubation, indicating a facultatively aerobic lifestyle.

Both strains shared several positive phenotypic characteristics, including catalase, oxidase, and *β*-galactosidase activities, nitrate reduction, glucose fermentation, esculin hydrolysis, and D-maltose assimilation, as well as negative reactions for Gram staining, hydrolysis of Tween 20 and Tween 80, and assimilation of capric acid and phenylacetic acid; these characteristics were consistent with those of closely related *Vibrio* type strains. However, several traits distinguished strains BS-M-Sm-2^T^ and MA40-2^T^ from their closest relatives, including differences in motility, the hydrolysis of casein, starch, and gelatin, and assimilation of D-glucose, D-mannose, and malic acid (Table 2).

Interestingly, although strains BS-M-Sm-2^T^ and MA40-2^T^ and their closest relatives all possessed glycoside hydrolase family 13 (GH13) genes (Fig. 3), strains BS-M-Sm-2^T^ and MA40-2^T^ exhibited starch-hydrolyzing activity, whereas *V. algarum* KACC 22588^T^ and *V. hannami* KACC 19277^T^ did not (Table 2). Detailed analysis of the GH13 repertoire showed that both novel strains commonly harbored endo-α-1,4-glucosidase (EC 3.2.1.1), endo-α-1,6-glucosidase (EC 3.2.1.68), and α-1,4-glucosidase (EC 3.2.1.20), which are key enzymes involved in starch degradation (Fig. 4B). In addition, strain BS-M-Sm-2^T^ possessed exo-α-1,4-glucobiosidase (EC 3.2.1.133) and pullulan endo-α-1,6-glucosidase (EC 3.2.4.41), whereas strain MA40-2^T^ harbored oligo-1,6-glucosidase (EC 3.2.1.10) as additional starch-degrading enzymes. In contrast, *V. algarum* KACC 22588^T^ encoded endo-α-1,4-glucosidase and α-1,4-glucosidase but lacked endo-α-1,6-glucosidase and the additional enzymes present in strains BS-M-Sm-2^T^ and MA40-2^T^, whereas *V. hannami* KACC 19277^T^ lacked all of these starch-cleaving enzymes. These differences in GH13 gene composition likely explain the observed variation in starch-hydrolyzing ability.

### Chemotaxonomic Characteristics

Ubiquinone-8 (Q-8) was identified as the sole respiratory isoprenoid quinone in strains BS-M-Sm-2^T^ and MA40-2^T^, consistent with the predominant quinone reported for members of the genus *Vibrio* [3, 4, 9]. The major cellular fatty acids (>5.0%) in both strains were C_16:0_, summed feature 3 (comprising C_16:1_ *ω*7*c* and/or C_16:1_ *ω*6*c*), and summed feature 8 (comprising C_18:1_ *ω*7*c* and/or C_18:1_ *ω*6*c*). In addition, strain BS-M-Sm-2^T^ contained C_12:0_ and C_14:0_ as major fatty acids. Overall, the fatty acid profiles of the two strains were similar to those of closely related *Vibrio* species; however, notable differences were observed in the composition of minor fatty acids (Table S4). For instance, C_17:0_ was detected in both BS-M-Sm-2^T^ and MA40-2^T^ but was absent or only present in trace amounts in the related type strains. Conversely, iso-C_14:0_ 3-OH was not detected in either strain but was present in the reference strains. The polar lipid profiles of both strains predominantly comprised PE, PG, and several unidentified lipids (Fig. S3), which is consistent with those of other *Vibrio* species [3, 4, 9]. In addition, strain MA40-2^T^ contained DPG, a feature commonly observed in many *Vibrio* species (Table 2).

### Conclusion of Taxonomic and Metabolic Analyses

The integration of polyphasic taxonomic evidence, including 16S rRNA gene- and whole-genome-based phylogenies, together with comprehensive physiological, biochemical, and chemotaxonomic characterizations, clearly demonstrates that strains BS-M-Sm-2^T^ and MA40-2^T^ represent two distinct novel species within the genus *Vibrio* (family *Vibrionaceae*, phylum *Pseudomonadota*). Accordingly, we propose the names *Vibrio phycocola* sp. nov. for strain BS-M-Sm-2^T^ and *Vibrio phycohabitans* sp. nov. for strain MA40-2^T^.

In addition to their taxonomic distinctiveness, the metabolic features of these strains indicate their potential ecological roles within the algal phycosphere. Both strains possess traits associated with nutrient provisioning, including vitamin biosynthesis and DNRA-mediated nitrogen retention, as well as mechanisms related to stress mitigation and microbial interactions. These characteristics suggest that they may contribute to algal growth promotion, the establishment of stable microbial communities, and enhanced resilience of the phycosphere. At the same time, differences in their metabolic capacities likely reflect functional diversification, enabling the two species to occupy distinct ecological niches in natural environments.

### Description of *Vibrio phycocola* sp. nov.

*Vibrio phycocola* (phy.co′co.la. Gr. neut. n. *phŷkos*, seaweed; L. suff. -*cola* (from L. masc./fem. n. *incola*), an inhabitant; N.L. masc. n. *phycocola*, an inhabitant of seaweed).

Colonies on MA are smooth and round. Cells are Gram-stain-negative, facultatively aerobic, motile rods with a polar flagellum. Growth occurs at 10–30°C (optimum, 25°C), at pH 6.0–10.0 (optimum, pH 7.0–8.0), and in the presence of 1.0–9.0% (w/v) NaCl (optimum, 2.0–3.0%). Catalase- and oxidase-positive. Nitrate is reduced to nitrite. D-Glucose fermentation and indole production are positive. Tyrosine, casein, starch, gelatin, and esculin are hydrolyzed, but Tween 20 and Tween 80 are not. Arginine dihydrolase, urease, and *β*-galactosidase activities are present. D-Glucose, L-arabinose, D-mannose, D-mannitol, D-maltose, and malic acid assimilated, whereas *N*-acetyl-glucosamine, potassium gluconate, capric acid, adipic acid, trisodium citrate, and phenylacetic acid are not. Q-8 is the sole respiratory quinone. The major cellular fatty acids (>5%) are C_12:0_, C_14:0_, C_16:0_, summed feature 3 (comprising C_16:1_ *ω*6*c* and/or C_16:1_ *ω*7*c*), and summed feature 8 (comprising C_18:1_ *ω*7*c* and/or C_18:1_ *ω*6*c*). The predominant polar lipids are PE, PG, an unidentified aminolipid, and two unidentified lipids.

The type strain is BS-M-Sm-2^T^ (=KACC 24066^T^ = DSM 119941^T^), isolated from the phycosphere of the marine brown alga *Sargassum miyabei* collected from a coastal region in South Korea. The genome size is 5,430 kb, and the genomic DNA G+C content is 44.2 mol%, as determined from the whole genome sequence. The GenBank accession numbers for the 16S rRNA gene and the genome sequences of strain BS-M-Sm-2^T^ are PQ311731 and CP176471–CP176472, respectively.

### Description of *Vibrio phycohabitans* sp. nov.

*Vibrio phycohabitans* (phy.co.ha?bi.tans. Gr. neut. n. *phŷkos*, seaweed; L. pres. part. *habitans*, inhabiting; N.L. part. adj. *phycohabitans*, seaweed-inhabiting).

Colonies on MA are smooth and circular. Cells are Gram-stain-negative, facultatively aerobic, motile rods with a polar flagellum. Growth occurs at 10–35°C (optimum, 25–30°C), at pH 6.0–9.0 (optimum, pH 7.0–8.0), and in the presence of 1.0–8.0% (w/v) NaCl (optimum, 2.0–3.0%). Catalase- and oxidase-positive. Nitrate is reduced to nitrite. D-Glucose fermentation is positive, whereas indole production is negative. Esculin, gelatin, casein, and starch are hydrolyzed, but tyrosine, Tween 20, and Tween 80 are not. Arginine dihydrolase and *β*-galactosidase activities are present, whereas urease activity is absent. D-Glucose, L-arabinose, D-mannose, *N*-acetyl-glucosamine, D-maltose, adipic acid, and malic acid are assimilated, whereas D-mannitol, potassium gluconate, capric acid, trisodium citrate, and phenylacetic acid are not. Q-8 is the sole respiratory quinone. The major cellular fatty acids (>5%) are C_16:0_, summed feature 3 (comprising C_16:1_ *ω*6*c* and/or C_16:1_ *ω*7*c*), and summed feature 8 (comprising C_18:1_ *ω*7*c* and/or C_18:1_ *ω*6*c*). The predominant polar lipids are PE, PG, DPG, and three unidentified lipids.

The type strain is MA40-2^T^ (=KACC 24064^T^ = DSM 119942^T^), isolated from the phycosphere of the marine red alga *Grateloupia divaricata* collected from a coastal region in South Korea. The genome size is 4,593 kb, and the genomic DNA G+C content is 39.8 mol%, as determined from the whole genome sequence. The GenBank accession numbers for the 16S rRNA gene and the genome sequences of strain MA40-2^T^ are PQ864772 and CP178627–CP178629, respectively.

## Supplemental Materials

Supplementary data for this paper are available on-line only at http://jmb.or.kr.



## Figures and Tables

**Table 1:** General genomic features of strains BS-M-Sm-2^T^ and MA40-2^T^ and their closely related type strains of the genus *Vibrio*.

Features^†^	1	2	3	4	5	6
Genome status^‡^ (no. of contigs)	C (2)	C (3)	D (3)	D (10)	C (4)	D (96)
Genome size (kb)	5,430	4,593	4,892	5,377	5,804	5,677
G+C content (mol%)	44.2	39.8	40.8	43.8	44.4	44.1
No. of total genes	4,819	4,136	4,573	5,059	5,204	5,128
No. of protein-coding genes	4,576	3,941	4,092	4,618	4,925	4,969
No. of total RNA genes	185	121	127	128	182	105
No. of rRNA genes	43	25	28	29	40	4
No. of tRNA genes	138	92	95	95	138	97
No. of non-coding RNA genes	4	4	4	4	4	4
No. of pseudogenes	58	74	354	313	97	54
No. of total CAZy^‡^ genes	84	67	71	76	87	83
Glycoside hydrolase	45	33	35	45	45	42
Glycosyltransferase	23	20	20	20	25	22
Polysaccharide lyase	3	8	9	7	3	5
Carbohydrate esterase	5	2	3	1	4	4
Carbohydrate-binding module	5	4	4	2	6	5
Auxiliary activity	3	0	0	1	4	5

Taxa: 1, strain BS-M-Sm-2^T^ (CP176471–2); 2, strain MA40-2^T^ (CP178627–9); 3, *V. algarum* KJ40-1^T^ (JAQLOI000000000); 4, *V. hannami* KACC 19277^T^ (JARQZP000000000). 5, *V. crassostreae* LMG 22240^T^ (AP025476–9); 6, *V. gigantis* LGP 13^T^ (MVJE00000000). The genomes of strains BS-M-Sm-2^T^ and MA40-2^T^ were sequenced in this study.

^†^General genomic features, except for CAZy genes, were retrieved from the GenBank database, whereas CAZy genes were identified and annotated using the dbCAN3 server integrated with the CAZy database [41].

^‡^C, complete; D, draft; CAZy, carbohydrate-active enzyme.

**Table 2:** Differential phenotypic characteristics between strains BS-M-Sm-2^T^ and MA40-2^T^ and their closely related type strains of the genus *Vibrio*.

Characteristic	1	2	3	4	5	6
Isolation source	Marine alga	Marine alga	Marine alga	Seawater	Oyster	Oyster
Colony colour	Cream	Cream	Light-yellow	Non-pigmented	Yellow	Cream
Anaerobic growth	+	+	+	–	+	+
Cell motility	+	+	–	+	+	+
Growth range of:						
Temperature (°C)	10–30	10–35	10–30	20–42	4–35	4–35
pH	6.0–10.0	6.0–9.0	6.0–10.0	6.0–10.0	na	na
NaCl (w/v, %)	1.0–9.0	1.0–8.0	1.0–8.0	2.0–8.0	2.0–6.0	2.0–6.0
Indole production*	+	–	–	–	–	–
Enzyme activity* of:						
Arginine dihydrolase	+	+	–	+	+	–
Urease	+	–	–	–	–	–
Hydrolysis* of:						
Casein, starch, gelatin	+	+	–	–	+	+
Tyrosine	+	–	+	–	+	+
Assimilation* of:						
D-Glucose, D-mannose, malic acid	+	+	−	−	+	+
L-Arabinose	+	w	−	+	w	−
D-Mannitol	+	−	−	−	+	+
*N*-Acetyl-glucosamine	−	+	−	−	+	+
Potassium gluconate	−	−	+	w	+	+
Adipic acid	−	w	−	−	−	−
Trisodium citrate	−	−	w	w	−	+
Major polar lipids^†^	PE, PG	PE, PG, DPG	PE, PG, DPG	PE, PG	na	na

Taxa: 1, strain BS-M-Sm-2^T^ (this study); 2, strain MA40-2^T^ (this study); 3, *V. algarum* KACC 22588^T^ [3]; 4, *V. hannami* KACC 19277^T^ [4]; 5, *V. crassostreae* LMG 22240^T^ [5]; 6, *V. gigantis* LMG 22741^T^ [6]. All strains are rod-shaped and positive for the following characteristics: nitrate reduction*, glucose fermentation*, enzyme activity* of catalase, oxidase, and *β*-galactosidase, hydrolysis* of esculin, and assimilation* of D-maltose. All strains are negative for the following characteristics: Gram-staining, hydrolysis* of Tween 20 and Tween 80, and assimilation* of capric acid and phenylacetic acid. Symbols: +, positive; –, negative; w, weakly positive; NA, not available.

*These analyses were conducted under the same conditions in this study.

^†^PE, phosphatidylethanolamine; PG, phosphatidylglycerol; DPG, diphosphatidylglycerol.
