# Correction to: *Vibrio phycocola* sp. nov. and *Vibrio phycohabitans* sp. nov., Isolated from the Phycosphere of Marine Algae

**DOI:** 10.4014/jmb.2026.3605.C05

**Published:** 2026-09-09

**Authors:** Jeong Min Kim, Byeong Jun Choi, Hülya Bayburt, Jae Kyeong Lee, Ju Hye Baek, Baolei Jia, Che Ok Jeon

**Affiliations:** 1 Department of Life Science, Chung-Ang University, Seoul 06974, Republic of Korea; 2 Xianghu Laboratory, Hangzhou 311231, P. R. China

The original article [1] described a novel *Vibrio* species isolated from the phycosphere of the marine brown alga *Sargassum miyabei* in May 2026, assigning strain BS-M-Sm-2^T^ the name *Vibrio phycocola*. Lyu and Xu [2] independently proposed the same name, *Vibrio phycocola*, for a different species (type strain WJH972^T^) in July 2026. However, during the validation process, the IJSEM List Editors received the documentation and request for validation of *Vibrio phycocola* Lyu and Xu 2026 before those for the name proposed by Kim *et al*. Under the ICNP, the sequence numbers assigned to names submitted for inclusion in a Validation List reflect the dates on which the validation requests in a form accepted for publication are received [3]. Therefore, *Vibrio phycocola* Lyu and Xu 2026 has priority, and validation of *Vibrio phycocola* Kim *et al*. 2026 cannot be approved.

To resolve this nomenclatural conflict, the corrected name should be *Vibrio sargassi* sp. nov. in place of the name *Vibrio phycocola* Kim *et al*. 2026 [1]. The type strain remains as originally designated: strain BS-M-Sm-2^T^ (= KACC 24066^T^ = DSM 119941^T^). The name *Vibrio phycohabitans* Kim *et al*. 2026 is unaffected by this correction.

## Description of *Vibrio sargassi* sp. nov.

*Vibrio sargassi* (sar.gas′si. N.L. gen. n. *sargassi*, of *Sargassum*, referring to the marine algal genus from which the type strain was isolated).

Colonies on MA are smooth and round. Cells are Gram-stain-negative, facultatively aerobic, motile rods with a polar flagellum. Growth occurs at 10–30°C (optimum, 25°C), at pH 6.0–10.0 (optimum, pH 7.0–8.0), and in the presence of 1.0–9.0% (w/v) NaCl (optimum, 2.0–3.0%). Catalase-and oxidase-positive. Nitrate is reduced to nitrite. D-Glucose fermentation and indole production are positive. Tyrosine, casein, starch, gelatin, and esculin are hydrolyzed, but Tween 20 and Tween 80 are not. Arginine dihydrolase, urease, and *β*-galactosidase activities are present. D-Glucose, L-arabinose, D-mannose, D-mannitol, D-maltose, and malic acid are assimilated, whereas *N*-acetyl-glucosamine, potassium gluconate, capric acid, adipic acid, trisodium citrate, and phenylacetic acid are not. Q-8 is the sole respiratory quinone. The major cellular fatty acids (>5%) are C_12:0_, C_14:0_, C_16:0_, summed feature 3 (comprising C_16:1_ *ω*6*c* and/or C_16:1_ *ω*7*c*), and summed feature 8 (comprising C_18:1_ *ω*7*c* and/or C_18:1_ *ω*6*c*). The predominant polar lipids are PE, PG, an unidentified aminolipid, and two unidentified lipids.

The type strain is BS-M-Sm-2^T^ (= KACC 24066^T^ = DSM 119941^T^), isolated from the phycosphere of the marine brown alga *Sargassum miyabei* collected from a coastal region in South Korea. The genome size is 5,430 kb, and the genomic DNA G+C content is 44.2 mol%, as determined from the whole-genome sequence. The GenBank accession numbers for the 16S rRNA gene and genome sequences of strain BS-M-Sm-2^T^ are PQ311731 and CP176471–CP176472, respectively.
