# Supplementary material for: Vibrio phycocola sp. nov. and Vibrio phycohabitans sp. nov., Isolated from the Phycosphere of Marine Algae
Source: J Microbiol Biotechnol. 2026 May 4;36:e2604007. doi: 10.4014/jmb.2604.04007 (PMC13189836; doi:10.4014/jmb.2604.04007)
Supplement: Supplementary file 1 [file jmb-36-e2604007-supple.pdf]

J. Microbiol. Biotechnol. 2026. 36: e2604007
https://doi.org/10.4014/jmb.2604.04007

Vibrio phycocola sp. nov. and *Vibrio phycohabitans* sp. nov., isolated from the phycosphere of marine algae

Jeong Min Kim[1], Byeong Jun Choi[1], Hülya Bayburt[1], Jae Kyeong Lee[1], Ju Hye Baek[1], Baolei Jia[2], and Che Ok Jeon[1,]*

[1]*Department of Life Science, Chung-Ang University, Seoul 06974, Republic of Korea*

[2]*Xianghu Laboratory, Hangzhou 311231, China*

*Author for correspondence: Che Ok Jeon (cojeon@cau.ac.kr)

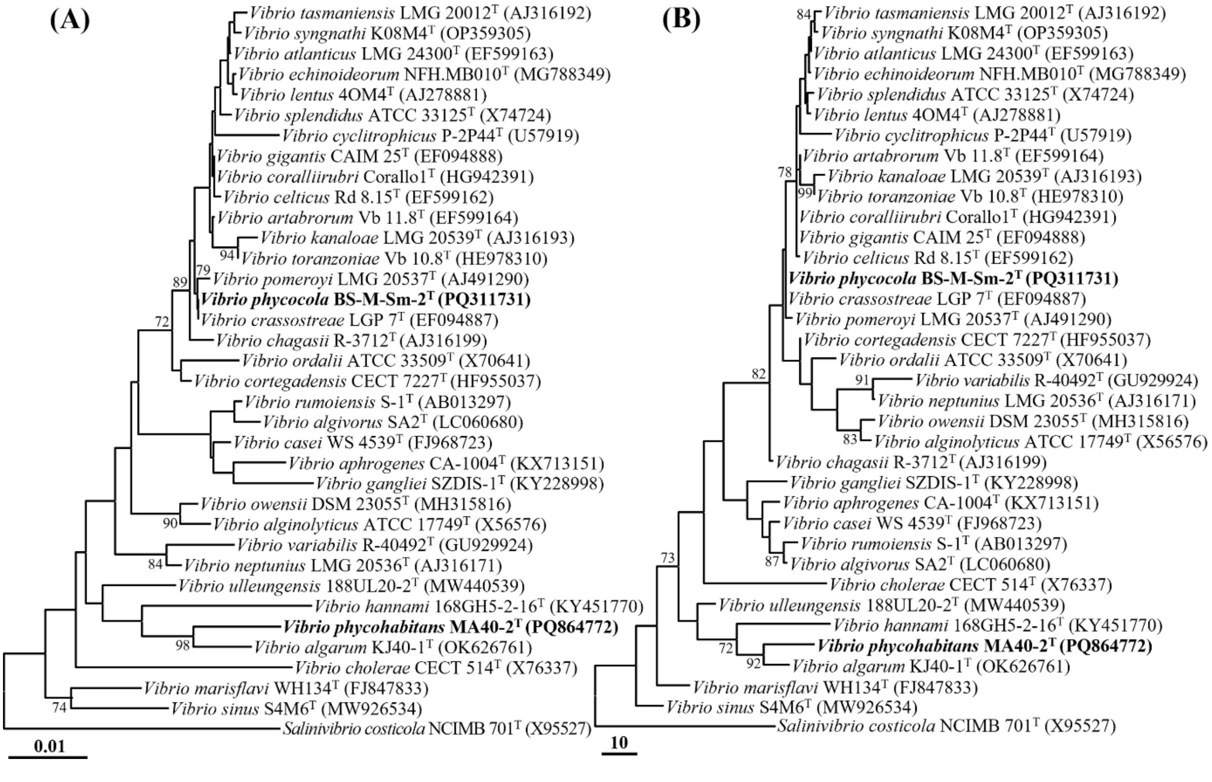

Fig. S1. Neighbor-joining (NJ; A) and maximum-parsimony (MP; B) phylogenetic trees showing the relationships of strains BS-M-Sm-2^{T} and MA40-2^{T} and their closely related taxa, based on 16S rRNA gene sequences. Bootstrap support values (>70%) are indicated at the nodes as percentages derived from 1,000 replicates. *Salinivibrio costicola* NCIMB 701^{T} (X95527) was used as the outgroup. Scale bars represent the number of nucleotide substitutions per site in the NJ tree and the number of character changes across the full 16S rRNA gene sequence in the MP tree.

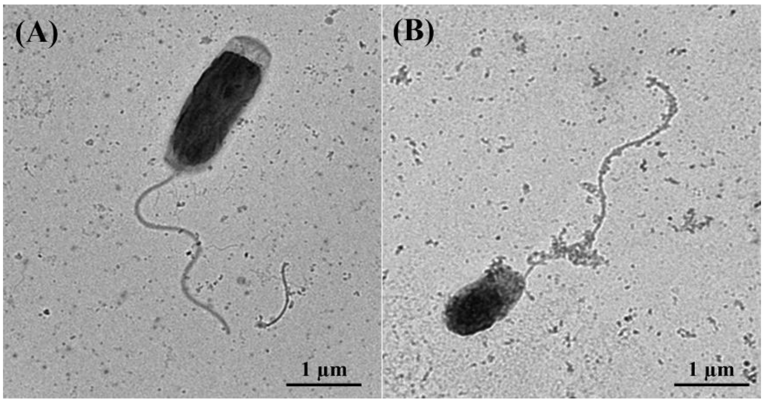

Fig. S2. Transmission electron micrographs showing the general morphologies of negatively stained cells of strains BS-M-Sm-2^T (A) and MA40-2^T (B), prepared using 2% (w/v) uranyl acetate after cultivation on marine agar at 25°C for 2 days. Scale bars, 1 μm.

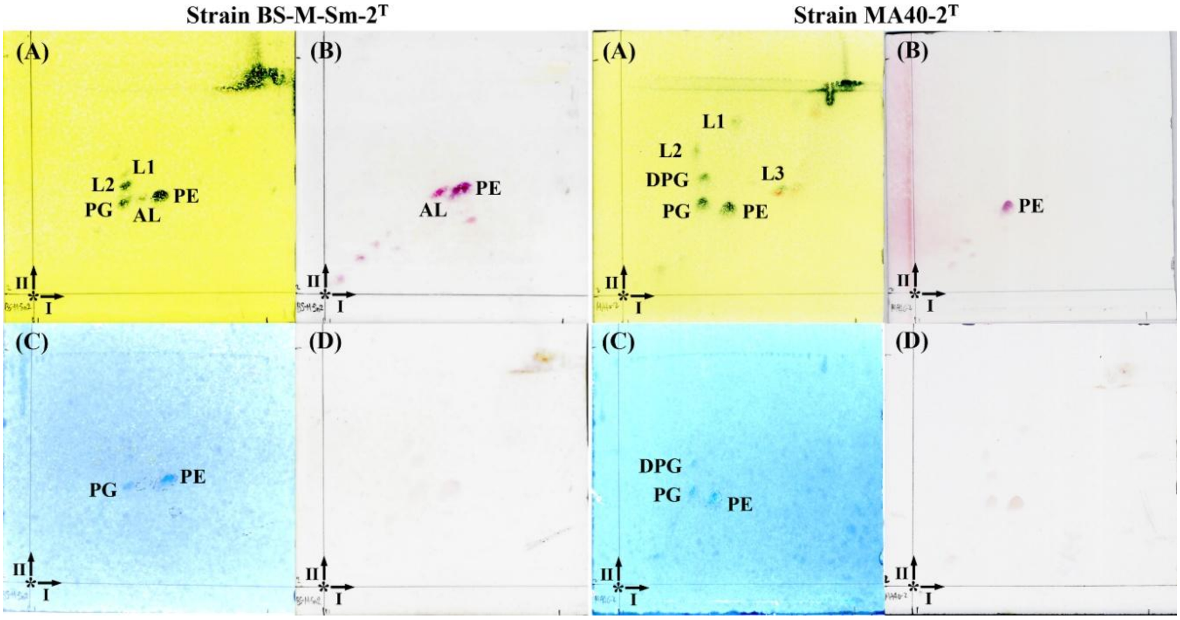

Fig. S3. Two-dimensional thin-layer chromatograms (TLC) showing the polar lipid profiles of strains BS-M-Sm-2^T and MA40-2^T. The solvent systems used were: (I) chloroform-methanol-water (65:25:4, v/v/v) and (II) chloroform-acetic acid-methanol-water (80:15:12:4, v/v/v/v). The TLC plates were sprayed with 10% ethanolic molybdophosphoric acid, ninhydrin, Dittmer-Lester, and *α*-naphthol/sulfuric acid reagents for the detection of total polar lipids (A), aminolipids (B), phospholipids (C), and glycolipids (D), respectively. Abbreviations: PE, phosphatidylethanolamine; PG, phosphatidylglycerol; DPG, diphosphatidylglycerol; AL, unidentified aminolipid; L, unidentified lipid.

Table S1. Potential ecological distributions of strains BS-M-Sm-2^T and MA40-2^T assessed by comparing their 16S rRNA gene sequences against metagenomic 16S rRNA amplicon datasets using the IMNGS platform, with a sequence similarity threshold of 99.0%.

Metagenome datasets	No. of datasets	Strain BS-M-Sm-2^T		Strain MA40-2^T	
		Prevalence (%)	ARA (%)	Prevalence (%)	ARA (%)
Crassostrea gigas	1,170	85.897	3.444	0.000	0.000
Eunicella cavoliniid	5	20.000	1.663	0.000	0.000
Litoditis marina	77	42.857	1.418	0.000	0.000
Tripneustes gratilla	26	100	0.939	42.308	0.207
Termite gut metagenome	155	9.032	0.937	0.000	0.000
Eunicella singularis	6	50.000	0.528	0.000	0.000
Oyster metagenome	319	15.674	0.415	0.000	0.000
Centricnemus leucogrammus	9	0.000	0.000	22.222	0.296
Jasus edwardsii	65	32.308	0.203	0.000	0.000
Coral metagenome	2,795	9.911	0.147	0.572	0.013
Annelid metagenome	37	18.919	0.153	0.000	0.000
Invertebrate metagenome	43	44.186	0.083	39.535	0.041
Algae metagenome	689	16.546	0.108	0.000	0.000
Solanum lycopersicum	10	0.000	0.000	20.000	0.081
Marine metagenome	37,438	8.863	0.036	1.060	0.031
Epibiont metagenome	132	11.364	0.065	0.000	0.000
Biofilm metagenome	2,953	2.370	0.056	0.000	0.000
Sponge metagenome	653	6.126	0.053	0.000	0.000
Fish gut metagenome	1,000	3.000	0.024	0.400	0.025
Aquatic metagenome	10,493	3.412	0.028	0.000	0.000
Microbial mat metagenome	720	2.500	0.027	0.000	0.000
Flotsam metagenome	25	16.000	0.024	0.000	0.000
Fish metagenome	706	8.215	0.021	0.000	0.000
Bacteriastrum furcatum	25	64.000	0.02	0.000	0.000
Marine plankton metagenome	151	13.245	0.02	0.000	0.000
Crioceris duodecimpunctata	10	0.000	0.000	70.000	0.019
Rock metagenome	169	4.142	0.016	0.000	0.000
Crustacean metagenome	219	1.370	0.015	0.000	0.000
Seawater metagenome	3,225	6.109	0.011	0.000	0.000

"Prevalence (%)" denotes the proportion of datasets containing sequences matching the 16S rRNA gene of each strain, whereas "ARA" (average relative abundance) represents the mean relative abundance of these sequences within the corresponding datasets. Habitats represented by fewer than three datasets or ARA values <0.01% for both strains across all datasets were excluded from the analysis.

Table S2. Genome relatedness among strains BS-M-Sm-2^T and MA40-2^T and their closely related type strains of the genus *Vibrio.*

		dDDH[†] value (%)					
		**1**	**2**	**3**	**4**	**5**	**6**
ANI[†] value (%)	**1**	–	23.2	22.5	23.6	44.3	41.7
	**2**	71.4	–	21.0	21.5	22.9	21.7
	**3**	71.3	75.6	–	20.8	22.6	21.8
	**4**	72.3	71.6	72.0	–	23.5	22.5
	**5**	91.6	71.3	71.3	72.2	–	39.2
	**6**	90.8	71.0	71.2	71.9	89.8	–

Taxa: 1, strain BS-M-Sm-2^T (CP176471–2); 2, strain MA40-2^T (CP178627–9); 3, *V. algarum* KJ40-1^T (JAQLOI000000000); 4, *V. hannami* KACC 19277^T (JARQZP000000000). 5, *V. crassostreae* LMG 22240^T (AP025476–9); 6, *V. gigantis* LGP 13^T (MVJE00000000).

[†]ANI, average nucleotide identity; dDDH, digital DNA-DNA hybridization.

Table S3. Identification of virulence-associated genes in strains BS-M-Sm-2^T and MA40-2^T and their closely related type strains within the genus *Vibrio*.

Category	Genes*	**1**	**2**	**3**	**4**	**5**	**6**
Exotoxin-related genes	*ctxAB*, *tdh*, *trh*, *vvhA*, *zot*, *tlh*	–	–	–	–	–	–
Toxin delivery	T3SS / T6SS loci	–	–	–	–	–	–
General secretion	T2SS loci	+	+	+	+	+	+
Adherence factors	*msh* / *pil* clusters	+	–	+	–	+	+
	ompU, *IlpA*, *mam7*	+	+	+	+	+	+
Motility or chemotaxis	*flg*, *fli*, *mot*, *che*	+	+	+	+	+	+

Taxa: 1, strain BS-M-Sm-2^T (CP176471–2); 2, strain MA40-2^T (CP178627–9); 3, *V. algarum* KJ40-1^T (JAQLOI000000000); 4, *V. hannami* KACC 19277^T (JARQZP000000000); 5, *V. crassostreae* LMG 22240^T (AP025476–9); 6, *V. gigantis* LGP 13^T (MVJE00000000).

* The genes presented in this table represent representative genes for each category previously reported in pathogenic or non-pathogenic *Vibrio* strains. Their detailed descriptions are as follows:

Exotoxin-related genes: *ctxAB*, cholera toxin subunits A and B; *tdh*, thermostable direct hemolysin; *trh*, TDH-related hemolysin; *vvhA*, *Vibrio vulnificus* hemolysin/cytolysin; *zot*, zonula occludens toxin; *tlh*, thermolabile hemolysin

Toxin delivery systems: T3SS loci, Type III secretion system loci; T6SS loci: Type VI secretion system loci

General secretion systems: T2SS loci: Type II secretion system loci

Adherence factors: *msh* cluster, mannose-sensitive hemagglutinin pilus gene cluster; *pil* cluster, pilus biogenesis/type IV pilus gene cluster; *ompU*, outer membrane protein U; *IlpA*, immunogenic lipoprotein A; *mam7*, multivalent adhesion molecule 7

Motility or chemotaxis genes: *flg*: flagellar basal body/flagellar biosynthesis genes; *fli*, flagellar assembly/flagellin-related genes; *mot*, flagellar motor protein genes; *che*, chemotaxis protein genes.

Table S4. Cellular fatty acid compositions (%) of strains BS-M-Sm-2^T and MA40-2^T and their closely related type strains of the genus *Vibrio*.

Fatty acid	1	2	3	4	5	6
Saturated:						
$C_{12:0}$	**7.1**	4.7	**5.9**	**5.2**	**7.2**	**5.6**
$C_{14:0}$	**7.0**	4.2	3.6	**7.9**	**5.6**	**5.2**
$C_{16:0}$	**26.6**	**10.1**	**17.5**	**13.2**	**19.1**	**20.2**
$C_{17:0}$	1.0	1.7	–	–	tr	tr
$C_{18:0}$	2.9	3.3	1.0	0.8	1.6	0.8
Unsaturated:						
$C_{14:1}$ *ω5c*	–	tr	0.7	tr	0.8	tr
$C_{16:1}$ *ω5c*	–	–	0.6	tr	–	–
$C_{17:1}$ *ω7c*	–	0.7	–	–	–	–
$C_{17:1}$ *ω8c*	0.5	–	–	tr	–	tr
$C_{18:1}$ *ω9c*	–	–	1.0	tr	1.2	–
$C_{18:3}$ *ω6c* (6,9,12)	–	0.6	–	–	tr	–
Branched:						
iso-$C_{10:0}$	0.5	0.9	1.0	0.7	0.9	0.5
iso-$C_{14:0}$	1.1	–	–	–	–	tr
iso-$C_{16:0}$	4.8	0.6	–	–	–	1.8
anteiso-$C_{17:1}$ A	–	–	0.5	tr	tr	–
iso-$C_{18:0}$	0.7	–	–	–	–	tr
iso-$C_{19:0}$	0.6	tr	1.9	1.4	1.1	1.4
Hydroxy:						
$C_{12:0}$ 3-OH	2.2	3.6	3.5	3.0	2.8	2.4
iso-$C_{12:0}$ 3-OH	1.0	1.0	0.7	0.6	tr	0.5
iso-$C_{14:0}$ 3-OH	0.9	–	–	–	–	tr
iso-$C_{15:0}$ 3-OH	–	0.5	1.6	–	0.8	tr
iso-$C_{17:0}$ 3-OH	–	–	1.5	0.9	0.7	0.7
Summed features*:						
1 (iso-$C_{15:1}$H and/or $C_{13:0}$ 3-OH)	0.9	0.9	0.9	0.6	–	tr
2 ($C_{14:0}$ 3-OH and/or iso-$C_{16:1}$ I	3.5	0.7	2.2	2.3	2.2	2.1
3 ($C_{16:1}$ *ω7c* and/or $C_{16:1}$ *ω6c*)	**26.6**	**40.7**	**44.4**	**44.9**	**43.4**	**43.9**
5 (anteiso-$C_{18:0}$ and/or $C_{18:0}$ *ω6,9c*)	–	–	–	1.3	–	–
7 ($C_{19:1}$ *ω7c* and/or $C_{19:1}$ *ω6c*)	1.9	–	–	–	–	tr
8 ($C_{18:1}$ *ω7c* and/or $C_{18:1}$ *ω6c*)	**7.2**	**23.1**	**10.2**	**13.6**	**9.2**	**9.1**

Taxa: 1, strain BS-M-Sm-2^T; 2, strain MA40-2^T; 3, *V. algarum* KACC 22588^T; 4, *V. hannami* KACC 19277^T; 5, *V. crassostreae* LMG 22240^T; 6, *V. gigantis* LMG 22741^T. All data were obtained from this study. Data are expressed as percentages for the total fatty acids, and fatty acids less than 0.5% in all strains are not indicated. Major components (>5.0%) are highlighted in bold. Symbols: tr, trace amount (<0.5%); –, not detected.

*Summed features are fatty acids that cannot be resolved reliably from other fatty acids using the chromatographic conditions chosen. The MIDI system groups these fatty acids together as one feature with a single percentage of the total.
